# Industrial Hemp and Tobacco as Multipurpose Crops: Traditional Uses, Contemporary Applications, and Future Perspectives

**DOI:** 10.3390/plants15182888

**Published:** 2026-09-21

**Authors:** Ivana Varga, Marinka Andraković, Patricia Crljić, Marta Đaković, Manda Antunović, Monika Tkalec Kojić, Dejan Agić

**Affiliations:** Faculty of Agrobiotechnical Sciences Osijek, Josip Juraj Strossmayer University of Osijek, 31000 Osijek, Croatiadakovicm@fazos.hr (M.Đ.); monikat@fazos.hr (M.T.K.);

**Keywords:** industrial hemp, tobacco, bioactive compounds, sustainability, regulation, human health

## Abstract

Industrial hemp (*Cannabis sativa* L.) and tobacco (*Nicotiana tabacum* L.) are two crops with long and important histories in human societies. This review aims to compare industrial hemp and tobacco from traditional uses to modern applications, focusing on bioactive compounds, health implications, and socio-economic and environmental aspects. Industrial hemp has traditionally been used for fibre, textiles, and paper and is now increasingly recognized as a multifunctional crop for food, cosmetics, pharmaceuticals, construction materials, and other bio-based industries. Tobacco, in contrast, has developed mainly as a commercial leaf crop and remains strongly associated with nicotine dependence and major public health concerns. Industrial hemp contains cannabinols, terpenes and antioxidant phenolics. These substances support hemp’s growing use in food, cosmetics, pharmaceuticals, construction materials, and other bio-based industries. In contrast, tobacco is dominated by alkaloids, especially nicotine, along with related compounds such as nornicotine and anatabine. Because of this, research on tobacco is mostly focused on addiction, toxic constituents, and major public health risks, while hemp research increasingly highlights potential biological activities and industrial benefits. However, hemp products still require strict quality control due to variability and the risk of THC contamination. Regulatory frameworks reflect these differences: tobacco products are tightly controlled globally due to proven health harms, while restrictions on industrial hemp cultivation have been gradually relaxing in many regions. Generally, the review shows two contrasting development paths—industrial hemp is increasingly positioned as a sustainable resource for the bioeconomy, whereas tobacco continues to raise serious health, environmental, and regulatory challenges.

## 1. Introduction

**Figure 1:**
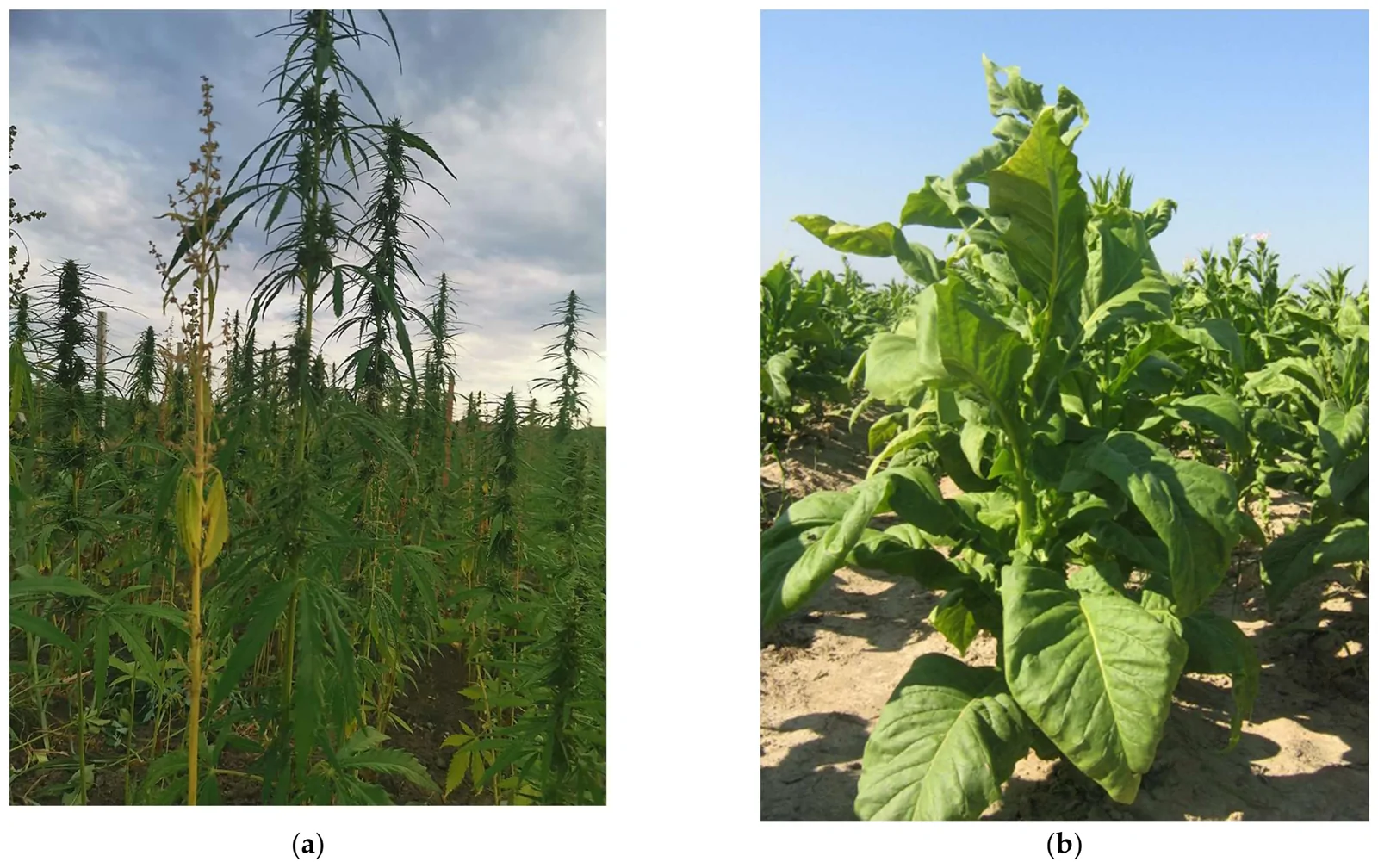
(**a**) *C. sativa* L.: yellow plants are male plants that have completed flowering, while green plants are female plants bearing seeds during the grain-filling (seed maturation) stage; (**b**) *N. tabacum* L.: American cigarette tobacco of the Virginia type (light, flue-cured).

Both industrial hemp (*Cannabis sativa* L.) and tobacco (*Nicotiana tabacum* L.) (Figure 1), have long been used throughout human history [1,2]. *C. sativa* L. has been traditionally cultivated for fiber, seed, and oil. Its fibers have been used in rope making and the textile and paper industry [3], while its seed and oils have served as a source of food and have been associated with potential medical efficacy [4]. Similarly, *N. tabacum* L. has a long presence in human culture and has been used for centuries not only in medicinal purposes [5] but also as a component of religious and ceremonial rituals [6]. Industrial hemp (Figure 1a) is an erect annual plant that can generally reach about 1–3 m in height, depending on its genotype and growing conditions. It is characterized by a slender, fibrous stem and palmately compound leaves with narrow, serrated leaflets [7]. Male plants are usually more slender and develop loose, branched clusters of pollen-producing flowers, whereas female plants are generally more robust and form denser inflorescences in which seeds develop [8,9,10]. Male plants of industrial hemp generally mature earlier than female plants [11]. Virginia-type tobacco (Figure 1b) is an erect annual plant that commonly reaches approximately 1–3 m in height under field conditions [12]. It is characterized by a strong, usually unbranched stem bearing large, simple leaves, while the upper part of the plant develops a terminal branched inflorescence with tubular, pinkish flowers [13].

Historically, tobacco and industrial hemp have received relatively limited scientific attention compared with many other economically important crops. However, research interest in these species has increased considerably in recent years. Recent research on tobacco has increasingly focused on the characterization of its chemical and bioactive constituents, including those present in seeds, leaves, and processing residues, as well as on their potential use in food, pharmaceutical, and antimicrobial applications, alongside the assessment of health risks associated with tobacco-derived products [13,14,15,16,17,18]. Research on industrial hemp has increasingly focused on its production potential and agronomic management, the chemical composition and nutritional value of seeds and other plant parts, interactions with pollinators, and the utilization of hemp biomass for biofuels and other value-added products [19,20,21,22,23]. Consequently, both industrial hemp and tobacco are increasingly being examined, not only as part of traditional culture but also as complex systems related to contemporary social problems [24].

Industrial hemp fiber is characterized by high strength, elasticity, durability, and water resistance. For that reason, it is used to produce ropes and cordage, sails and canvas, fabrics, clothing and footwear, tarpaulins, tents, fishing nets, firefighting hoses and equipment, bags and sacks, horse harness gear, etc. [24,25,26]. Male plants contain a higher percentage of fiber, and their fiber is generally of better quality than that of female plants.

After the fiber is extracted, a woody part of the stem remains. This material is used in the manufacture of paper and cellulose, as an insulating material, and it can also be used as fuel [27]. Hemp seed contains more than 30% oil, from which edible oil can be produced for human consumption [28]. Because the oil dries easily, it is also used in the production of paints and varnishes. The seed is additionally used as feed for poultry and birds. Seed yields can be around 1.5–2 t/ha [29].

The woody portion of the hemp stem is also utilized for producing various specialty papers, such as cigarette paper, security and banknote paper, Bible paper, greaseproof paper, fine art paper, insulating strips for electrical capacitors, special nonwoven paper, and filter paper, including coffee filters and tea bags [30]. Industrial hemp fibers are also used in automotive manufacturing, for example, in brake components and interior linings [31].

Tobacco has been used since ancient American civilizations for ritual and medicinal purposes, and its global spread accelerated after Europeans encountered it in the 16th century, especially through the promotion of Jean Nicot, whose name is reflected in Nicotiana and nicotine. Today, tobacco (plants of the genus Nicotiana, family Solanaceae) is widely cultivated and processed into products such as cigarettes, cigars, snuff, and chewing tobacco, and nicotine has also been used in pesticides and some medical applications [32,33,34]. Many reported pharmacological and traditional uses are linked to nicotine’s action on nicotinic receptors, influencing multiple body systems and leading to a wide range of claimed effects (e.g., antimicrobial, analgesic, antispasmodic, and wound-related applications) [35,36,37]. Tobacco leaves contain nicotine as the main alkaloid, along with other alkaloids and various phytochemicals (organic acids, glycosides, flavonoids, and terpenic compounds) that contribute to their biological activity [38,39]. Fatica et al. [40] investigated the biomass of *N. tabacum* L. cv. Solaris (2016–2018) to assess its chemical composition and suitability for alternative uses beyond smoking tobacco. The results suggest that Solaris biomass could be useful for animal nutrition and is well-suited for anaerobic digestion, showing good fiber/protein characteristics, low total alkaloids, and promising biomethane potential.

Previous reviews have generally discussed industrial hemp and tobacco separately, focusing on specific aspects such as agronomy, chemical composition, health effects, or industrial applications. Therefore, the aim of this review is to compare industrial hemp (*C. sativa* L.) and tobacco (*N. tabacum* L.) within a common framework, considering their traditional uses, botanical and agronomic characteristics, chemical composition, health and safety aspects, industrial applications, and legal regulation. Particular attention is given to their contrasting development pathways, with industrial hemp increasingly associated with sustainable production and new bio-based applications, while tobacco remains strongly linked to health concerns and strict regulation. By considering both crops together, this review also aims to identify current research gaps and possible directions for future studies.

## 2. Literature Search Strategy

A narrative literature search was conducted using the Web of Science Core Collection, Scopus, PubMed, and Google Scholar databases. The final search was performed in September 2026 and included publications available up to the date of the search, without a strict lower publication-date limit because historical sources were also relevant to the scope of the review. Representative search terms included “Cannabis sativa”, “industrial hemp”, “hemp”, “*Nicotiana tabacum*”, and “tobacco”, combined with terms such as “agronomy”, “traditional use”, “chemical composition”, “bioactive compounds”, “health effects”, “industrial applications”, “sustainability”, “economic importance”, “market”, and “regulation”. Peer-reviewed original research articles and review papers directly relevant to the topics covered in this review were prioritized. Authoritative institutional, statistical, and regulatory sources were additionally consulted for information related to production, markets, legislation, and regulatory frameworks. Publications were selected according to their relevance to the comparative evaluation of industrial hemp and tobacco, while duplicate records and studies outside the scope of the review were excluded. As this study was designed as a narrative review, no formal systematic-review protocol was applied.

## 3. Botanical and Agronomic Characteristics

### 3.1. Taxonomy of Tobacco and Industrial Hemp

*C. sativa* L. is an annual plant belonging to the *Cannabaceae* family. Cannabaceae is a relatively diverse plant family comprising approximately 170 species [41]. It is usually dioecious, although monoecious varieties also occur. Industrial hemp refers to cultivars containing less than 0.3% THC, which are cultivated for their fiber or seeds. This distinguishes industrial hemp from hemp used for psychoactive purposes. Within *C. sativa* L., two subspecies are recognized: *C. sativa* subsp. *sativa* and *C. sativa* subsp. *indica* [42].

The genus *Nicotiana*, in the family *Solanaceae*, includes approximately 75 species, mainly native to the Americas. Only two species are cultivated, resulting from the hybridization of wild species, one of which is *N. tabacum* L. [43]. It is an annual or short-lived perennial plant, primarily cultivated for its nicotine-rich leaves [1]. It includes more than 1600 cultivars, but there are three commonly cultivated types, which differ in agronomic and marketable properties: Flue-cured (Virginia), Burley, and Oriental [44].

### 3.2. Morphology and Cultivation Requirements

Industrial hemp can grow in various climates [2]. It develops a strong root with many lateral roots, which helps the plant absorb water and nutrients efficiently [14]. Industrial hemp is characterized by rapid growth and a strong vegetative mass [45]. The plant has a deep and branched root system that improves soil structure and increases drought resistance [46]. The stem is strong and fibrous and can reach a height of 2 to 5 m, depending on the variety and growing conditions [47]. The leaves are palmately divided, and the plant is usually dioecious, with separate male and female plants [45]. It prefers a temperate climate with optimal temperatures between 16 and 27 °C [47]. It grows best on deep, well-drained soils with a neutral to slightly alkaline pH [46]. Hemp requires relatively few pesticides because it is naturally resistant to many pests and diseases [45]. Dense seeding enables effective weed control without the use of herbicides [47]. The cultivation of industrial hemp is considered ecologically sustainable due to the low input and wide applicability of the biomass [46].

Tobacco, on the other hand, prefers warm and moderately humid conditions with a long growing season [14]. Its optimal growth temperature is usually between 20 and 30 °C. The leaves are large, oval to lanceolate, rich in nicotine and other alkaloids, and represent the main commercial part of the plant [15]. The stem is erect and branched, and the flowers are pink to reddish in color, collected in terminal inflorescences [16]. Tobacco prefers warm and moderately humid climates with a long growing season. The optimal temperature for growth ranges between 20 and 30 °C [14]. The plant requires well-drained, fertile soils rich in nitrogen, potassium, and phosphorus [15]. Tobacco cultivation often involves intensive use of fertilizers and pesticides, which can have negative environmental consequences [16]. Harvesting is carried out manually or mechanically, most often by gradually removing the leaves as they mature [14]. After harvesting, the leaves undergo a drying and fermentation process that significantly affects the chemical composition and quality of the final product [15].

According to Small and Cronquist [42], there are recognized two main groups of *Cannabis*: *sativa* and *indica*. The sativa group can be found in northern regions and is characterized by a higher content of fiber and oil, along with a low intoxicant potential. The other group, *indica*, is often found in southern regions and has a higher intoxicating potential. In practice, *C. sativa* subsp. *sativa* is mostly associated with industrial hemp, with a THC content of <0.3%, and cultivated for fiber, seed, and oil. Opposite, *C. sativa* subsp. *indica* is mostly the psychoactive type with higher THC content. However, this is not always the case, as there are different hybrid types, and the chemical composition of THC/CBD is not strictly tied to the species and subspecies classification. Modern legal frameworks classify cannabis based on THC content rather than botanical taxonomy. Different varieties of tobacco have been developed through selection to meet specific requirements for taste, chemical composition, and mode of consumption [48]. The most widespread commercial species is *N. tabacum*, while *Nicotiana rustica* is used less frequently due to its significantly higher nicotine content [42]. Varieties of Virginia tobacco are characterized by a high sugar content and are used primarily for the production of cigarettes [49]. Burley tobacco has a low sugar content, but a high ability to absorb aromas, which is why it is often used in cigarette blends [48]. Oriental tobacco has small leaves and an intense aroma and is grown mainly in Mediterranean regions for specialized tobacco products [50].

## 4. Historical Use and Cultural Significance

Industrial hemp has been found on Neolithic sites in Central Asia. According to Chris Duvall [51], in northern East Asia, cannabis was the earliest plant used for textile manufacturing. Although the species origin is in Asia, the oldest physical evidence was found in Europe: a hemp rope in the Czech Republic dating back to 26,900 BC. It is therefore believed that hemp arrived in Europe very early and was primarily used for rope manufacturing. The first major use of hemp dates back to China, where it served as a source of fiber for textiles and paper. By the 17th century, the plant spread to America, and in some periods its cultivation was even mandatory [2]. The seeds provided a source of food, stems were used for fastening objects, and cloth and paper were made [51]. According to archaeological findings of tobacco seeds, people in North America were using the plant 12,300 years ago [1]. Civilizations such as the Mayas and Aztecs incorporated tobacco smoke into religious and ritual practices, often using pipes, while northern Indigenous nations used tobacco in medical and social rituals [5]. These practices highlight the importance of tobacco prior to European contact. It spread to Europe in the 15th century through European explorers [52]. During the 16th century, its use spread rapidly to Africa and Asia, mostly due to the expansion of global trade routes and colonial networks.

By the 17th and 18th centuries, a smoking culture had developed throughout Europe, and tobacco cultivation became economically and socially very significant. The Industrial Revolution of the 19th century led to the mechanical production of cigarettes and the wider availability of tobacco. As the habit of smoking became more widespread, early scientific research began to document the association between tobacco use and adverse health outcomes. By the late 19th and early 20th centuries, the medical literature increasingly linked smoking to respiratory diseases, including lung cancer, marking the first appearance of scientific concern about tobacco use [53].

Although modern industrial hemp refers to low-THC cultivars grown primarily for fiber and seeds, historical sources document the ritual and ceremonial use of Cannabis sativa, prior to today’s taxonomic and regulatory restrictions. Ritual use helps us understand why regulation came later. One of the earliest notes on ritual use of provided by Herodotus, [54], who described the ancient Scythian peoples’ use of hemp in funerary and purification rituals. According to Herodotus [54], participants of a funeral inhaled cannabis smoke to purify the spirit. In Hindu tradition, cannabis held a sacred status and became associated with the god Shiva. Ritual use included the oral consumption of plants for smoking of flower buds, practices that continue in certain religious contexts to this day [6]. In various African and Asian societies, cannabis was part of ceremonies aimed at connecting with spirits and ancestors [55]. In some parts of Europe, particularly in Poland and Ukraine, sources describe traditional rituals associated with the harvesting of hemp. These practices included beliefs that a special procedure marks the beginning of the season and that the plant is capable of warding off evil spirits or ensuring good fortune [56]. Tobacco played a far more pronounced ceremonial and ritual role among native Americans than it would later assume in Europe and the modern world. Native American cultures considered tobacco, which was smoked, chewed, drunk, and even snorted, a medium of communication with the supernatural world, often mediated by shamans. In Mesoamerican societies, including Maya, tobacco itself was revered as a divine plant [57].

Archaeological evidence further supports that ancient Mesoamericans used tobacco in healing and ceremonial rituals. The plant was not only smoked but also prepared as liquid infusions. In their culture, tobacco is associated with healing or communication with spirits [58]. Tobacco was introduced to Europe in the late fifteenth century through European travelers who transported both the plant and knowledge of its use. Initially, tobacco entered European contexts embedded within social and ceremonial practices, although its ritual significance gradually changed as its use spread [52].

## 5. Production and Economic Relevance

Industrial hemp and tobacco represent economically important crops but differ substantially in the structure and development of their global markets. Industrial hemp is increasingly associated with diversified value chains based on seeds, fibres, textiles, food products, cosmetics, construction materials, biocomposites, and other bio-based products. International trade statistics, however, considerably underestimate the economic importance of hemp because many processed hemp products are not represented by dedicated Harmonized System codes. When national product classifications were considered in addition to UN Comtrade data, the estimated value of recorded hemp-product exports in 2022 increased to approximately USD 213 million [59]. Canada was particularly important in hemp-seed exports; France, Spain, and the Netherlands were major exporters of raw and semi-processed hemp products, while China represented an important market for hemp yarn and textile products [60].

The tobacco market remains considerably larger and more established, although global raw tobacco production has shown a long-term declining trend. According to the 2025 WHO Framework Convention on Tobacco Control report [60], China, India, and Brazil were the three largest producers of raw tobacco in 2023, with China alone producing almost as much as the following nine largest producers combined. International trade nevertheless remains economically important: global trade in raw tobacco reached approximately USD 22.1 billion in 2023. Brazil was the leading exporter of raw tobacco, followed by Zimbabwe, the United States, and India, whereas China remained an important producer, importer, and manufacturer. Trade values for conventional raw and manufactured tobacco products have remained relatively stable over the past decade, while newer product categories, including heated tobacco and electronic nicotine-delivery products, have shown increasing trade values.

**Figure 2:**
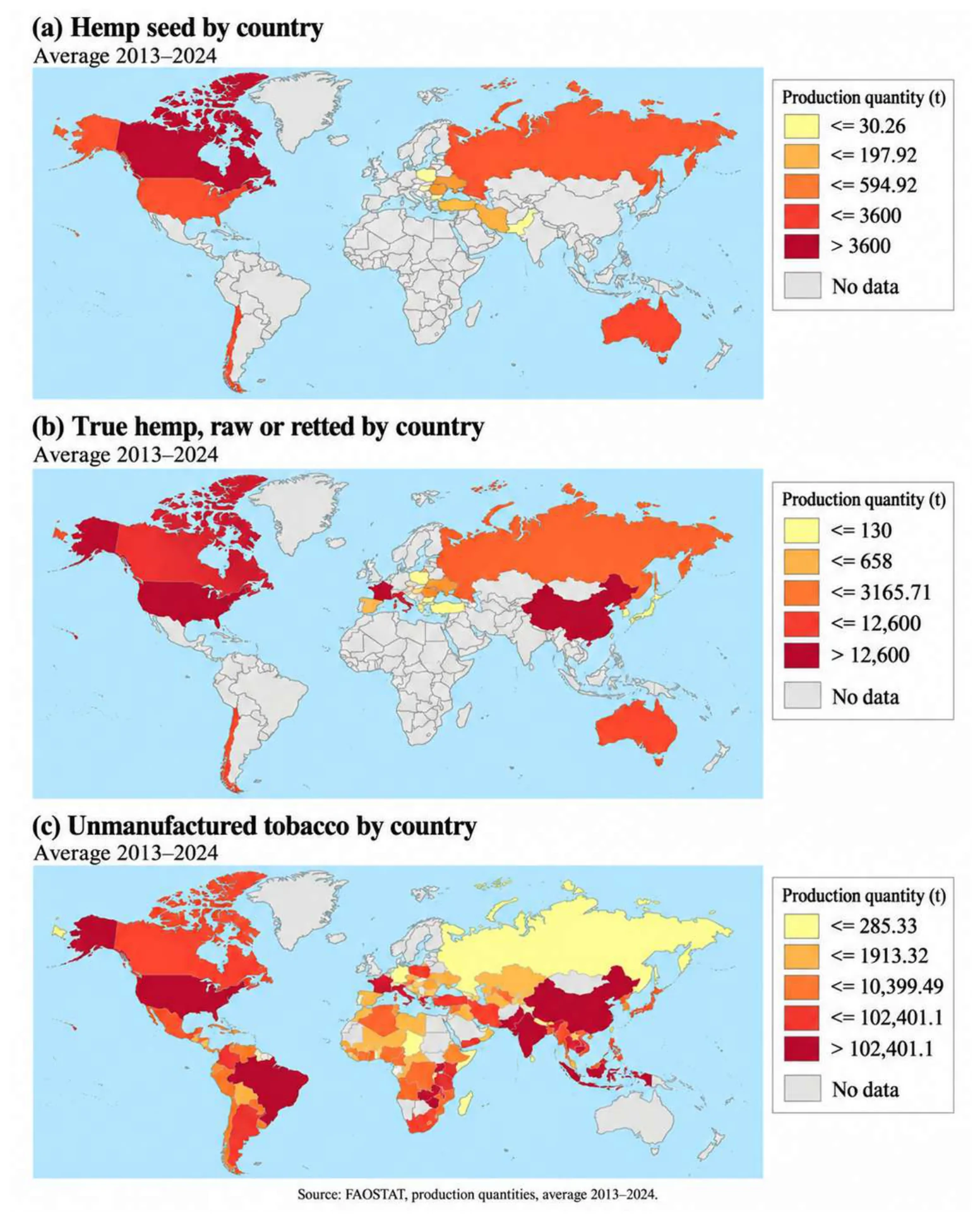
Average production quantities (t) by country for (**a**) hemp seed, (**b**) true hemp, raw or retted, and (**c**) unmanufactured tobacco during 2013–2024 [61].

Based on FAOSTAT data [61] averaged over the 2013–2024 period, clear differences were observed in both the scale and geographical distribution of hemp and tobacco production (Figure 2). For hemp seed production (Figure 2a), Canada was the most prominent producer, being the only country in the highest production class, with an average annual production exceeding 3600 t. The United States, Russia, Australia, and Chile were among the countries in the next production class, with average production between approximately 595 and 3600 t.

The production of true hemp, raw or retted (Figure 2b), showed a different geographical pattern. The United States, China, and France belonged to the highest production category, each exceeding an average of 12,600 t year^−1^ during 2013–2024. Several other countries, including Russia, Australia, Italy, and some Central and Eastern European countries, showed intermediate production levels.

In contrast, unmanufactured tobacco production was considerably more widespread and occurred at a much larger scale (Figure 2c). China, India, Brazil, and the United States were among the major producing countries, each falling within the highest production class of more than 102,401 t year^−1^. Tobacco production was also substantial across parts of Southeast Asia, South America, and southern and eastern Africa. Overall, the maps demonstrate that hemp production remains concentrated in a relatively small number of countries, whereas tobacco production is geographically broader and characterized by substantially higher production volumes.

These two crops show contrasting market trajectories. Industrial hemp is developing through diversification into new food, material, construction, and bioeconomy applications, although its global market remains difficult to quantify accurately. Tobacco retains a much larger and highly internationalized market, but its traditional production base is gradually contracting in many regions under increasing public-health regulation. At the same time, tobacco production and trade are becoming relatively more important in several low- and middle-income regions, particularly in Africa.

## 6. Chemical Composition and Bioactive Compounds

### 6.1. Major Groups of Compounds

The chemical complexity of both *C. sativa* L. and *N. tabacum* is defined by several major classes of secondary metabolites that determine their biological and ecological functions [62].

According to Rawat and Mali [38], the methanolic leaf extract of *N. tabacum* showed significant, dose-dependent antinociceptive (pain-relieving) activity in Wistar mice, increasing pain reaction/latency times and reducing acetic-acid-induced writhing, with effects comparable to a standard reference drug.

**Figure 3:**
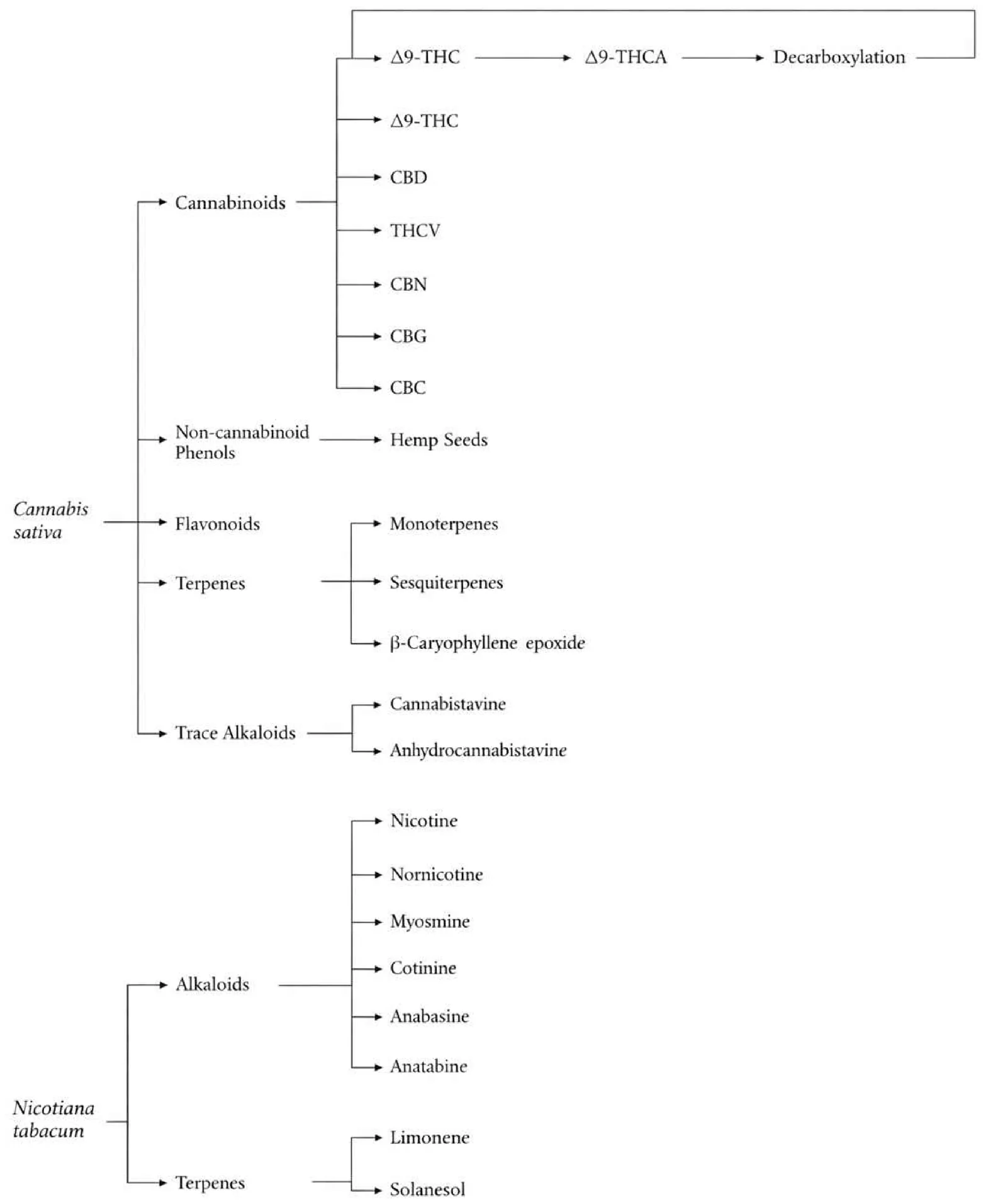
Major groups of biologically active compounds in *C. sativa* and *N. tabacum.* Abbreviations: Δ9-THC, delta-9-tetrahydrocannabinol; Δ9-THCA, delta-9-tetrahydrocannabinolic acid; CBD, cannabidiol; THCV, tetrahydrocannabivarin; CBN, cannabinol; CBG, cannabigerol; CBC, cannabichromene.

In *C. sativa*, more than 500 chemical constituents have been reported, among which approximately 125 compounds have been identified as cannabinoids [63,64]. Cannabinoids represent a unique group of C21 terpeno-phenolic compounds that are considered chemotaxonomically specific to the genus *Cannabis* [63]. In addition to cannabinoids, cannabis contains a diverse array of non-cannabinoid phenols, flavonoids, terpenes, and trace alkaloids, contributing to its complex phytochemical profile [27]. Padilla-González et al. [28] used untargeted metabolomic approaches to demonstrate that hemp seeds possess an exceptionally diverse metabolome, including multiple classes of secondary metabolites and a newly identified molecular family broadly distributed across different *C. sativa* varieties. Beyond the two most well-known cannabinoids, Δ9-tetrahydrocannabinol (THC) and cannabidiol (CBD), several other major cannabinoids have been identified in *C. sativa*, including tetrahydrocannabivarin (THCV), cannabinol (CBN), cannabigerol (CBG), and cannabichromene (CBC) [26,27]. Cannabinoids are synthesized predominantly in the glandular trichomes, specialized resin-secreting epidermal structures located on female inflorescences [27]. Structurally, cannabinoids possess a characteristic C21 terpenophenolic backbone and, notably, do not contain nitrogen, distinguishing them from alkaloids despite their pronounced psychoactive properties [26]. Among cannabinoid constituents, Δ9-THC is the principal psychoactive compound and occurs naturally in the plant primarily as Δ9-tetrahydrocannabinolic acid (Δ9-THCA), which undergoes decarboxylation upon heating [25,26]. In contrast, CBD is non-psychoactive and has attracted significant pharmacological interest due to its anti-epileptic and neuroprotective properties [25,29]. Figure 3 provides a schematic overview of the major classes of secondary metabolites identified in *C. sativa* and *N. tabacum*, illustrating differences in their phytochemical composition.

In contrast to cannabis, the biological activity of *N. tabacum* is largely driven by alkaloids, particularly nicotine, which underlies the plant’s strong addictive potential [25]. However, although nicotine is the primary addictive agent, tobacco dependence is increasingly viewed as a multifactorial process shaped by additional bioactive compounds [30]. Tobacco smoke contains several minor alkaloids—including nornicotine, myosmine, cotinine, anabasine, and anatabine—which, like nicotine, interact with nicotinic acetylcholine receptors and may act synergistically to reinforce addiction [30,31]. While nicotine is present at approximately 10 mg per cigarette, these minor alkaloids typically occur at microgram-per-cigarette levels [31]. By comparison, alkaloids are relatively rare in *C. sativa*, with only two spermidine alkaloids—cannabisativine and anhydrocannabisativine—having been identified, highlighting fundamental phytochemical differences between cannabis and tobacco [26].

Terpenes represent another important class of bioactive compounds in both species and are largely responsible for their characteristic aroma. In cannabis, more than 120 terpenes have been identified, including monoterpenes and sesquiterpenes, which contribute to ecological interactions such as herbivore deterrence and may also modulate cannabinoid activity. Certain terpenes, such as β-caryophyllene epoxide, are considered characteristic markers of cannabis and are used by drug-detection animals [27]. In tobacco smoke, terpenes such as limonene and solanesol have been linked to neuroprotective and antioxidant properties, although their biological relevance in smokers remains under investigation [25].

A comparison of biologically active substances in industrial hemp and tobacco highlights two chemically distinct plant species whose biological effects are largely determined by the composition of their secondary metabolites. As noted by Twaij and Hasan [25], secondary metabolites—such as alkaloids, phenolics, terpenes, and cannabinoids—play a key role in plant defense and largely shape the physiological impact of plant-derived products. Frazzini et al. [32] reported that hulled industrial hemp seeds contain a complex mixture of bioactive molecules, including phenolic acids, flavonoids, and bioactive peptides, whose combined antioxidant activity supports their classification as functional ingredients.

An especially diverse phytochemical profile characterizes industrial hemp. Radwan et al. [27] report that *C. sativa* contains more than 500 identified chemical constituents, including a unique group of cannabinoids classified as C21 terpenophenolic compounds, synthesized mainly in glandular trichomes. The most prominent cannabinoids include Δ9-tetrahydrocannabinol (THC) and cannabidiol (CBD), while other cannabinoids such as cannabigerol (CBG) and cannabichromene (CBC) also contribute to the plant’s biological activity [27]. In contrast, the chemical profile of tobacco is dominated by alkaloids, particularly nicotine, which functions as the plant’s principal defensive compound. West and Cox [30] emphasize that nicotine is responsible for tobacco’s addictive properties and represents the major alkaloid accumulated in tobacco leaves. According to Zhang et al. [33], tobacco also contains a range of other bioactive constituents, including phenolic compounds, although alkaloids remain the dominant metabolite group. Figure 3 provides a schematic comparison of the major classes of biologically active compounds identified in *C. sativa* and *N. tabacum*, highlighting the predominance of cannabinoids in hemp and alkaloids in tobacco.

Phenolic composition further differentiates these two species. Industrial hemp seeds and their by-products are recognized as valuable sources of antioxidant phenolic compounds. Leonard et al. [34] demonstrate that hempseed hulls are particularly rich in phenylpropionamides, including lignanamides such as cannabisins A and B, which exhibit strong antioxidant activity. Similarly, Benkirane et al. [35] show that the total phenolic content of hemp seeds is highly dependent on extraction conditions. In tobacco, phenolic compounds such as chlorogenic acid and rutin are important contributors to antioxidant capacity, as reported by Zou et al. [12].

Current research indicates clear biochemical differences between hemp and tobacco, reflecting their distinct ecological roles and applications. While hemp is increasingly explored for nutritional, pharmaceutical, and industrial purposes, tobacco research remains largely focused on alkaloid biosynthesis, addiction mechanisms, and health-related effects [62]. A comparative overview of the principal biologically active compounds in tobacco and cannabis, including their primary targets and biological effects, is presented in Table 1.

### 6.2. Links Between Chemical Composition and Biological Effects

The biological effects of plants are directly determined by their chemical composition, with *C. sativa* L. representing a multifunctional crop whose value arises from both its secondary metabolites and structural constituents [73]. Industrial hemp produces a wide range of biologically active compounds, including cannabinoids, terpenes, and phenolic compounds, which contribute to its ecological interactions, industrial performance, and potential biomedical relevance [73,74]. Cannabinoids are a distinctive class of metabolites unique to Cannabis, and although industrial hemp varieties are characterized by low tetrahydrocannabinol (THC) content, these compounds remain important from a regulatory, agronomic, and breeding perspective [74]. Advances in hemp genome editing further underscore the importance of understanding cannabinoid biosynthesis pathways, as targeted genetic modifications may allow the optimization of metabolite profiles for industrial and pharmaceutical applications [37]. Floares et al. [38] demonstrated that phenolic compounds extracted from *C. sativa* seeds are the primary drivers of antimicrobial activity and can enhance the effectiveness of conventional antibiotics, directly linking chemical composition with biological function. Beyond its biochemical properties, hemp is also valued for its structural and material characteristics, which are closely tied to its chemical composition. Hemp stems contain a high proportion of cellulose and hemicellulose, which confer exceptional tensile strength and durability to bast fibers, making hemp a sustainable alternative to conventional construction and insulation materials. The favorable chemical composition of hemp fibers contributes to a low environmental footprint, biodegradability, and suitability for use in eco-friendly building systems [65]. From an agronomic perspective, hemp cultivation generally requires relatively low inputs, shows strong weed suppression, and improves soil structure, further supporting its classification as a sustainable industrial crop [36].

In contrast, the biological effects of tobacco are largely dominated by alkaloids, particularly nicotine, which plays a central role in the plant’s defense mechanisms and its socio-economic impact. Tobacco cultivation and post-harvest processing—especially curing practices—substantially influence nicotine concentration and the overall chemical composition of cured leaves. Traditional tobacco-curing methods are associated with significant environmental pressures, including deforestation, greenhouse gas emissions, and increased energy consumption, as well as socio-economic challenges in tobacco-growing regions [40]. Taken together, these contrasting chemical and biological characteristics highlight fundamental differences between hemp and tobacco, positioning hemp as a versatile and sustainable industrial crop, whereas tobacco remains primarily associated with health risks and environmental concerns [39,40].

A comparative overview of the main botanical, agronomic, historical, and phytochemical characteristics of industrial hemp and tobacco is presented in Table 2.

### 6.3. Modern Analytical Approaches for Phytochemical Characterization

Modern analytical techniques have significantly improved the characterization of bioactive compounds in industrial hemp and tobacco. HPLC and UPLC are widely used for the separation and quantitative analysis of cannabinoids, alkaloids, phenolic compounds, and other non-volatile metabolites. In *C. sativa*, these methods are particularly important for the accurate determination of THC, THCA, CBD, CBDA, and minor cannabinoids, while coupling liquid chromatography with mass spectrometry (LC-MS/MS or high-resolution MS) allows the identification of compounds present at much lower concentrations [75,76]. Mass spectrometry has also expanded cannabis research from targeted cannabinoid determination toward untargeted phytochemical profiling, providing a more complete view of the metabolome and helping to relate minor cannabinoids, terpenes, and phenolic compounds to their potential biological and pharmacological activities [77].

In tobacco, LC-MS/MS, GC-MS, and multi-platform metabolomics have been successfully applied to characterize nicotine and minor alkaloids, phenolic compounds, flavonoids, amino acids, lipids, and other metabolites. These approaches have revealed substantial variation according to cultivar, developmental stage, leaf position, and curing process. Importantly, advanced metabolomic profiling can help distinguish compounds associated with tobacco toxicity and dependence from other phytochemicals with potential biological activity [78]. For example, LC-MS/MS metabolomics identified numerous flavonoids in tobacco leaves and linked individual compounds with different inflammatory responses [79]. Therefore, combining chromatographic, spectrometric, and metabolomic approaches provides a stronger connection between phytochemical composition, biological effects, quality control, and the evaluation of potential pharmaceutical or nutraceutical applications.

## 7. Medicinal and Therapeutic Applications

Historically, both tobacco and industrial hemp were integrated into traditional medical systems, although their roles and cultural meanings differed markedly. Tobacco was used extensively by Indigenous cultures in the Americas long before its global commercialization, serving ritual, spiritual, and therapeutic functions. It was employed as an analgesic, antiseptic, and respiratory aid; leaves and infusions were applied to wounds, bites, and inflammatory conditions, and smoke was sometimes used ceremonially to treat illness or to ward off perceived spiritual causes of disease. These practices reflect a holistic medical framework in which tobacco was regarded as both a physical and symbolic agent of healing [41].

Industrial hemp—particularly low-THC, fiber-type *C. sativa* L.—also has a long history of use in traditional medicine across Asia, the Middle East, and parts of Europe. In Ayurvedic and traditional Chinese medicine, hemp seeds and extracts were used to support digestion, relieve pain, reduce inflammation, and promote overall vitality. Hemp seed preparations were commonly consumed as tonics, while topical applications were used for skin conditions and joint discomfort. Unlike tobacco, hemp’s medicinal role was more closely linked to its nutritional value and calming physiological effects than to psychoactive potential [42,43].

Modern biomedical research has fundamentally reshaped the perception of tobacco. While traditional uses emphasized its healing potential, contemporary science focuses on its toxicological profile and the health risks associated with its consumption. Studies have identified nicotine as the primary biologically active compound responsible for addiction, cardiovascular stimulation, and neurological effects. Additionally, tobacco processing and consumption, particularly through smoking, generate a wide range of harmful substances, including tobacco-specific nitrosamines, aldehydes, and polycyclic aromatic hydrocarbons. As a result, tobacco is now viewed primarily as a public health concern rather than a medicinal resource [80,81].

In contrast, industrial hemp has gained increasing scientific attention as a source of potentially beneficial bioactive compounds. Research highlights cannabidiol (CBD) as a major non-psychoactive cannabinoid with broad pharmacological properties, including anti-inflammatory, antioxidant, neuroprotective, and anxiolytic effects. Studies emphasize hemp’s interaction with the endocannabinoid system, which plays a role in regulating pain, immune response, and neurological function. Modern scientific understanding, therefore, positions industrial hemp as a promising botanical resource for functional foods, nutraceuticals, and pharmaceutical development [45,46].

The therapeutic potential of industrial hemp is largely attributed to its rich profile of cannabinoids, fatty acids, and phenolic compounds. Hemp-derived CBD has been widely studied for its potential in the management of chronic pain, epilepsy, anxiety disorders, and inflammatory conditions. Unlike psychoactive cannabinoids, CBD does not produce intoxication, which supports its suitability for a broad range of clinical and wellness applications. Evidence suggests that CBD may modulate neurotransmitter systems and immune-related pathways, contributing to its neuroprotective and anti-inflammatory properties [45,46].

Hemp seed oil represents another important therapeutic product and is valued for its balanced omega-3 to omega-6 fatty acid ratio, which may support cardiovascular health, skin integrity, and metabolic function. The oil is commonly used in dietary supplements and cosmetic formulations due to its moisturizing, anti-inflammatory, and antioxidant effects. In addition, hemp fibers are gaining attention for biomedical uses, including wound dressings and sustainable medical textiles, owing to their strength, biodegradability, and biocompatibility. Collectively, these diverse applications emphasize industrial hemp’s role as both a medicinal resource and a functional industrial crop [47].

Although tobacco once held a prominent role in traditional medicine, its modern therapeutic use is highly restricted. Today, the main medicinal application of tobacco-derived compounds is the controlled use of nicotine in smoking-cessation therapies. Nicotine replacement products—such as patches, gums, and inhalers—are intended to reduce withdrawal symptoms and dependence on combustible tobacco products. These pharmaceutical uses reflect a harm-reduction strategy rather than a direct therapeutic use of tobacco itself [48].

Beyond cessation therapies, nicotine has also been investigated for potential neurological effects, including possible roles in cognitive function and research on neurodegenerative diseases. However, such applications remain tightly regulated and largely experimental because of nicotine’s addictive properties and associated health risks. Consequently, tobacco’s historical status as a medicinal plant has largely been replaced by its modern classification as a source of controlled pharmacological substances and a public health concern [41,44,49]. Although tobacco is no longer regarded as a medicinal plant in contemporary clinical practice, several tobacco-derived compounds have been examined for potential neuroactive and neuroprotective effects under controlled experimental conditions. Selected examples are summarized in Table 3.

## 8. Industrial and Economic Applications

### 8.1. Hemp: Textiles, Biocomposites, Food Products, Cosmetics

Industrial hemp is widely recognized as a multi-purpose crop with applications across numerous industrial sectors. Its long, strong fibers are used in the production of textiles, ropes, paper, insulation materials, and construction composites. Hemp-based biocomposites are increasingly employed in the automotive and building industries due to their lightweight properties, durability, and reduced environmental footprint compared to synthetic materials. The high cellulose content of hemp fibers makes them particularly suitable for sustainable manufacturing and biodegradable product development [50,51].

In the food and cosmetic industries, hemp seeds and oil are valued for their nutritional and functional properties. Hemp-based food products include protein powders, flours, plant-based beverages, and edible oils, which are marketed for their high-quality amino acid profiles and essential fatty acids. In cosmetics, hemp oil is incorporated into skin and hair care products for its moisturizing, anti-inflammatory, and antioxidant effects. These combined industrial uses position hemp as a key crop in the growing bioeconomy and circular production systems [52].

### 8.2. Tobacco: Agriculture and Pharmaceutical Use (Nicotine Replacement Therapies)

Tobacco remains a major global agricultural commodity, cultivated in more than 100 countries and supporting millions of farmers and workers, particularly in developing economies. It plays a significant role in rural livelihoods and international trade, with large-scale production systems emphasizing leaf quality, curing practices, and supply-chain efficiency. Despite declining smoking rates in some regions, tobacco continues to generate substantial economic value through domestic markets and export networks [53].

In the pharmaceutical sector, tobacco’s economic relevance also extends beyond conventional cigarette manufacturing. Nicotine extracted from tobacco plants serves as a key raw material for nicotine replacement therapies and for emerging alternative nicotine-delivery systems. These products represent a growing segment of the health and wellness market, shaped by public health initiatives aimed at reducing smoking-related harm. This dual role of tobacco as both an agricultural and pharmaceutical resource reflects an ongoing transition in its industrial identity [54].

### 8.3. Economic Relevance and Market Trends

The global industrial hemp market has expanded rapidly, driven by rising demand for sustainable materials, plant-based nutrition, and natural wellness products. Market analyses point to increasing investment in hemp-based bioplastics, green construction materials, and functional foods. Policy changes in several regions have further encouraged hemp cultivation as part of environmental and economic sustainability strategies, highlighting its potential roles in carbon sequestration, soil remediation, and rural development [51].

By contrast, the tobacco industry is facing stricter regulation, higher taxation, and changing consumer preferences, particularly in high-income countries. Although conventional cigarette markets are contracting, tobacco’s overall economic footprint remains substantial due to diversification into reduced-risk products, pharmaceutical nicotine, and international trade. This contrast reflects a broader economic transition: industrial hemp is increasingly linked to future-oriented, sustainable markets, whereas tobacco is adapting to mounting regulatory and public health pressures within a shifting global economy [55,56].

## 9. Health Impacts and Safety Considerations

### 9.1. Beneficial and Adverse Effects

Research on tobacco and nicotine products points to a complex interplay between perceived benefits and well-established harms. Qualitative evidence indicates that many people who use tobacco products report short-term perceived benefits, including temporary relaxation, enhanced social interaction, subjective weight control, and relief of cravings or stress effects linked to nicotine’s pharmacological actions on the central nervous system. However, these perceived benefits coexist with substantial negative consequences across multiple domains of health and functioning. People who smoke frequently report reduced physical performance and exercise capacity, increased fatigue, and adverse emotional responses such as guilt, anxiety, or depression related to their use and its risks. Together, these findings suggest that perceived positive effects may obscure deeper health and psychosocial harms that often become more evident over time or during cessation attempts [57].

Crucially, adverse outcomes extend well beyond self-reported perceptions when tobacco use persists or is combined with other nicotine products. Epidemiological and clinical evidence show that tobacco and nicotine use can produce a wide range of signs and symptoms, including impaired oral hygiene, chronic cough, and physiological damage affecting multiple organs and systems. These harms involve not only physical deterioration but also functional impairment, influencing cognitive, emotional, and social dimensions of health among people who use tobacco products. Generally, these outcomes reinforce that any alleged short-term benefits do not offset long-term risks, particularly in light of chronic disease pathways and declines in health-related quality of life [57]. These long-term consequences are summarized in Table 4, which provides a system-based overview of symptoms and diseases associated with tobacco consumption.

Industrial-hemp-derived cannabidiol (CBD) products are widely used because they are non-psychoactive and are often marketed for wellness benefits; nevertheless, available evidence indicates that adverse effects are a significant concern. Surveys, case reports, meta-analyses, and post-marketing safety assessments have documented negative outcomes associated with CBD product use, including gastrointestinal discomfort and sensations of feeling “high,” the latter likely resulting from contamination with psychoactive Δ^9^-tetrahydrocannabinol (THC). Although CBD itself is not intoxicating, the study notes that many commercial products contain THC levels that exceed safe thresholds, increasing the risk of unintended psychotropic effects. Additional toxicological concerns identified in experimental studies include potential liver toxicity and other dose-dependent physiological effects. Moreover, inconsistent product quality, inaccurate labeling, and the absence of regulatory approval under European Novel Food regulations may increase consumer risk, underscoring the need for standardized testing and strict regulatory oversight to ensure the safe use of hemp-derived products [58].

### 9.2. Health Risks Associated with Tobacco Use

The health risks associated with tobacco use are well-established and span cardiovascular, respiratory, infectious, metabolic, and oral diseases. Cigarette smoking is a cause of atherosclerosis, contributing to clinical cardiovascular events through chronic endothelial dysfunction and accelerated plaque formation [100]. Tobacco smoke’s toxic complex, containing thousands of chemicals, contributes to vascular inflammation, oxidative stress, and immune compromise, substantially increasing the likelihood of conditions such as coronary heart disease, stroke, and other major cardiovascular endpoints.

Respiratory systems are particularly vulnerable to tobacco exposure. In addition to increasing the risk of chronic obstructive pulmonary disease (COPD), tobacco smoke accelerates the decline in lung function and heightens susceptibility to infectious respiratory illnesses such as pneumonia [60]. Smoking also disrupts lung development in adolescents and hastens deterioration in pulmonary mechanics in adults, contributing to greater long-term morbidity. Periodontitis and diseases of the oral mucosa are likewise causally associated with smoking, with large observational datasets confirming a strong dose-dependent relationship [50]. Moreover, the use of smokeless tobacco in different regions shows mixed—but in some settings significant—associations with cancer and cardiovascular outcomes, indicating substantial regional variation in health risks depending on product type and composition [59].

### 9.3. Safety Profile of Industrial Hemp

The safety profile of industrial hemp (*C. sativa* L., low-THC cultivars) differs substantially from that of tobacco products because of its distinct nutritional and biochemical composition. Reviews of hemp seeds and seed-derived products emphasize their nutritional quality and potential health functionality, highlighting high levels of unsaturated fatty acids, proteins, minerals, and other bioactive compounds [61]. These constituents may support nutritional supplementation and contribute to anti-inflammatory potential in the diet, and there is growing interest in their possible role in dietary strategies aimed at preventing inflammatory and chronic-degenerative diseases [61].

However, the safety profile of hemp-derived compounds such as cannabidiol (CBD) must be clearly distinguished from the beneficial nutritional attributes of whole seeds. Evidence from the World Health Organization’s critical review of cannabidiol suggests that, although CBD is generally well tolerated, adverse effects—including drowsiness, gastrointestinal disturbances, and potential drug interactions—have been reported, particularly at higher doses and in clinical contexts [62]. Regulatory and market analyses further point to concerns about inconsistent quality control, inaccurate labeling, and the availability of unauthorized or insufficiently regulated CBD products, which may expose consumers to unintended THC levels or contaminants [51]. In addition, systematic evaluations of CBD products on the European market report instances of non-compliance with food safety requirements and the absence of pre-market authorization, raising further concerns regarding consumer safety and product standardization [58]. Collectively, these findings indicate that while industrial hemp products can offer meaningful nutritional value, hemp-derived extracts require rigorous regulatory oversight, standardized testing, and transparent labeling to ensure safety and purity.

### 9.4. Quality Control and Product Standardization

Quality control and standardization are essential for ensuring product safety and consumer protection, particularly given the substantial variability in composition across both tobacco and hemp-derived product markets. Tobacco regulatory science highlights the need for transparent and reproducible risk-assessment approaches to support regulatory decision-making, taking into account not only chemical hazards but also patterns of use, marketing influences, and differential responses across populations [63]. Without rigorous risk-assessment frameworks, regulatory authorities cannot reliably estimate population-level risks or potential benefits associated with modified tobacco products.

In the context of industrial hemp and CBD products, the lack of harmonized regulatory standards has created major challenges related to product classification, safety evaluation, and market authorization. Regulatory assessments indicate that many hemp-derived products—especially CBD-containing foods and supplements—fall under novel food classification requirements and may enter the market without consistent pre-market safety approval or standardized testing protocols [51]. Evidence from the World Health Organization further underscores the need for formal quality benchmarks, noting that variability in product composition, labeling accuracy, and cannabinoid concentrations can complicate safety assessments and risk communication [62]. These regulatory gaps contribute to inconsistent safety profiles and raise concerns about contamination, unintended THC exposure, and consumer misinformation, highlighting the importance of robust quality-control systems, transparent labeling, and internationally aligned regulatory frameworks to protect public health.

The major differences between industrial hemp and tobacco regarding health implications, industrial applications, sustainability, and regulatory context are summarized in Table 5.

## 10. Legal and Regulatory Framework

### 10.1. Legal Status of Industrial Hemp and Tobacco

As tobacco became increasingly popular in the 20th century, numerous studies highlighted its health risks. Warner and Tam [101], show that evidence of smoking’s association with lung cancer, cardiovascular disease, and premature mortality directly prompted governments to introduce regulatory measures. Adopted in 2003, the WHO Framework Convention on Tobacco Control (WHO FCTC) [102] obligates signatory countries to implement measures to reduce tobacco use, such as advertising bans and restrictions on public smoking.

Tobacco control laws vary among countries; in most, cultivation is legal but subject to state supervision [103]. Most countries set the legal age for tobacco purchase at 18, but some, such as the USA, have raised it to 21 [104]. Most states have banned smoking in indoor public spaces, based on scientific evidence of the harmful effects of secondhand smoke on health [103]. The emergence of new tobacco and nicotine products, such as e-cigarettes, challenges existing regulations, as their long-term health effects are not yet fully understood [102]. Tobacco regulation involves an ongoing conflict between public health goals and the economic interests associated with production, taxation, and international trade [105].

Unlike tobacco, there is no single binding contract for industrial hemp, and in most countries, hemp with a THC content below 0.3% is legal for cultivation and processing because it has no psychoactive effects and poses little risk to public health [45]. Research also highlights its potential in sustainable agriculture, carbon emissions reduction, soil phytoremediation, and the production of environmentally friendly materials [46]. The legal framework originates from the UN Single Convention on Narcotic Drugs of 1961, [106], which included hemp but did not distinguish it from psychoactive cannabis. Later interpretations allowed member states to grow hemp for industrial or scientific purposes [107]. Although partly harmonized through international guidelines, legislation on industrial hemp varies between countries. In the EU, cultivation is legal but highly regulated, with the permitted THC threshold recently increased from 0.2% to 0.3% in 2023 [108].

In the United States, the 2018 Farm Bill legalized industrial hemp, defining it as a plant with less than 0.3% THC. In many states, cultivation requires a license, field monitoring, and laboratory testing. In the United States, industrial hemp itself is not generally subject to a federal age restriction; however, hemp-derived consumer products, particularly cannabinoid-containing products, may be subject to age restrictions and other regulatory requirements depending on the product type and state or local legislation [109].

### 10.2. International Regulatory Differences

A comparison of tobacco and industrial hemp clearly shows the differences in regulatory logic. Tobacco is regulated globally primarily from a public health perspective, as scientific evidence confirms its harmfulness. This has resulted in restrictive measures such as high taxation, advertising bans, and restrictions on consumption in public spaces, in line with the WHO FCTC. Industrial hemp, on the other hand, is regulated primarily through drug control and agricultural policy, rather than as a public health issue. While the regulatory framework for tobacco tends to focus on reducing consumption and protecting the population, contemporary trends in industrial hemp are moving towards deregulation and encouraging production, particularly due to its economic and environmental benefits. Industrial hemp is thus increasingly taking the place of a strategic agricultural and industrial crop, while tobacco remains the subject of global regulation aimed at its gradual limitation.

Regulatory frameworks for industrial hemp and tobacco differ substantially across global markets because the two crops are regulated for fundamentally different reasons. Industrial hemp is generally governed through agricultural, narcotics-control, food, and product-safety legislation, with legal status often determined by jurisdiction-specific THC thresholds. Tobacco, in contrast, is predominantly regulated through public-health legislation aimed at controlling production, commercialization, advertising, access, and consumption. These differences are particularly evident in the United States, the European Union, China, and Canada, where distinct approaches are applied to cultivation, processing, permitted uses, and product commercialization. The main regulatory characteristics of these jurisdictions are summarized in Table 6.

## 11. Implications for Pharmaceutical, Food, and Agricultural Industries

### 11.1. Pharmaceutical Industry

Industrial hemp is an important raw material for the production of cannabidiol (CBD), one of ab125 identified cannabinoids in hemp plants, which are being investigated for their potential therapeutic effects in the treatment of chronic pain, anxiety, epilepsy and inflammatory conditions [117,118]. The pharmaceutical industry uses CBD from industrial hemp in the development of drugs for certain medical indications, with the only currently widely approved CBD drug being Epidiolex^®^, used for treating two rare and severe forms of epilepsy: Lennox–Gastaut and Dravet syndrome [119].

Industrial hemp is not only a source of cannabinoids for pharmaceutical products, but scientific literature also highlights the potential application of secondary metabolites from hemp in various biomedical and therapeutic contexts [120]. Existing business and financial relationships between pharmaceutical and tobacco companies may create conflicts of interest. These include pressures to reduce the promotion of pharmaceutical smoking cessation products to protect tobacco industry profits, as documented in historical analyses of business records from both sectors [121]. The tobacco industry historically opposed early forms of nicotine cessation therapy, but later began to develop and market such products themselves to maintain control of the nicotine market and respond to regulation [122].

### 11.2. Food Industry

Unlike industrial hemp, tobacco does not play a functional role in food chains or contribute to the development of sustainable food products. Industrial hemp seeds, on the other hand, are nutritionally rich, containing significant amounts of protein, essential fatty acids, and dietary fiber, and are increasingly used as an ingredient in the production of foods and dietary supplements [123]. Hemp oil and hemp flour from hemp seeds are used in food products such as cold-pressed salad oil, protein powders, and baking mixes, making industrial hemp a significant source of healthy plant nutrients [124,125].

**Figure 4:**
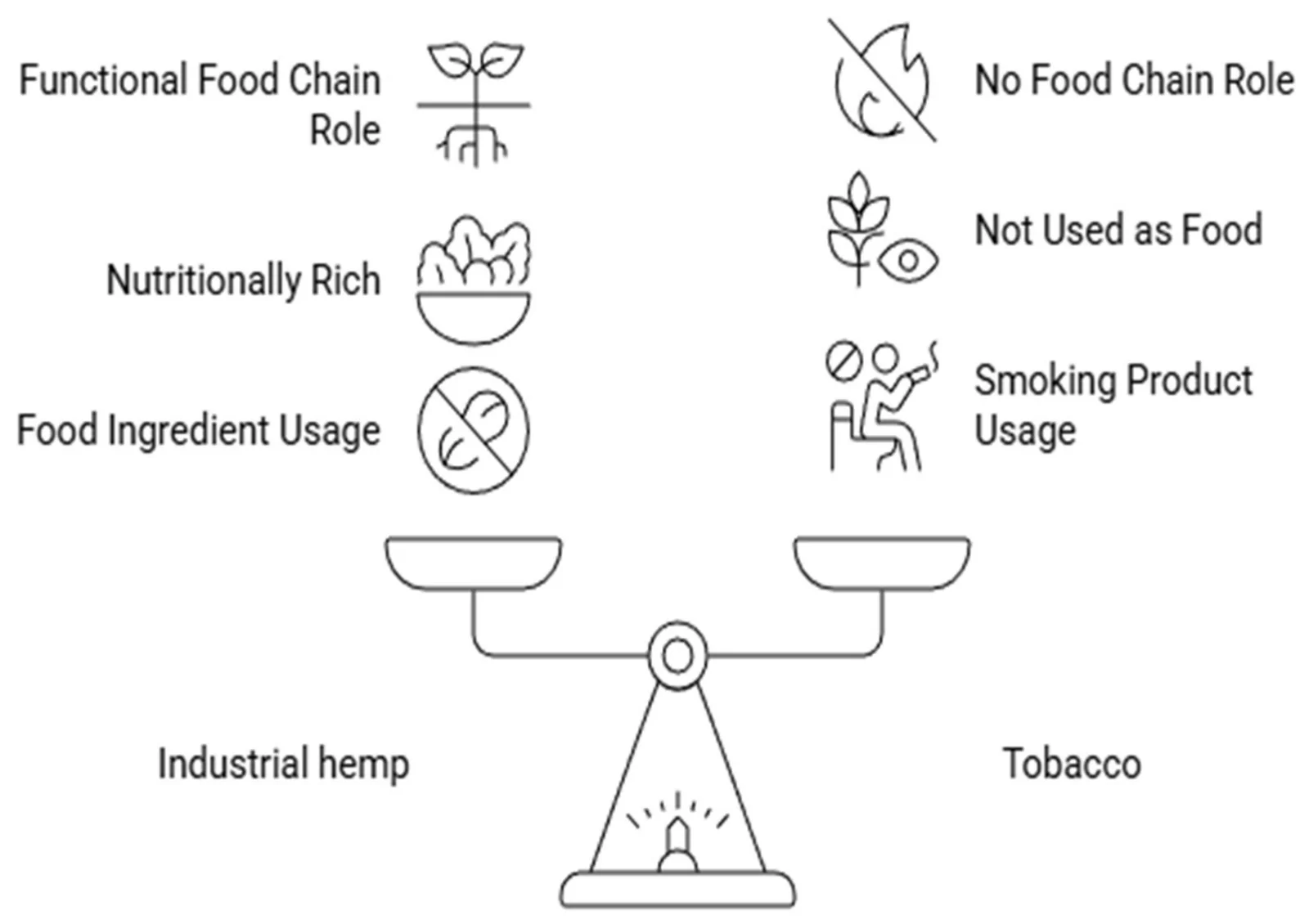
Comparison between hemp and tobacco uses.

Tobacco is not traditionally used as food in the food industry. The leaves of the tobacco plant are processed almost exclusively for products intended for smoking, chewing or snorting, and are not used as food [48]. Although the tobacco leaf naturally contains certain constituents, such as sugars and other organic compounds, these are studied in the context of tobacco smoke and its toxicology, and are not used in safe food products [49]. Accordingly, Figure 4 illustrates a comparison between industrial hemp and tobacco with respect to their roles and applications in the food industry, highlighting the nutritional value and food-use potential of hemp in contrast to the absence of tobacco from food production.

### 11.3. Agricultural Industry

Industrial hemp is a highly sustainable crop. It grows quickly, requires few pesticides and fertilizers, and has a wide range of applications. The agricultural industry considers hemp a strategic crop that combines environmental benefits with economic viability [46]. Production of industrial hemp has increased significantly in recent years, with an over 80% rise in seven years. Hemp fibers are used in textiles as a sustainable and robust alternative to traditional fibers such as cotton. Hemp seeds and oil are used in the food and nutritional industry due to their high protein, omega-3 fatty acid, and essential nutrient content. The construction industry uses hemp stalks to make hemp concrete, insulation, and other sustainable building materials [47].

In the European Union, tobacco cultivation has been declining in recent decades, but it remains an important crop [47]. Its primary agricultural use is the production of leaves for cigarettes, cigars, and other tobacco products [126]. Tobacco cultivation generates substantial agronomic and industrial waste, including roots, stems, and leaf ribs, which can be used for extracting bioactive compounds or as inputs in other agricultural sectors [127].

## 12. Current Research and Future Directions

### 12.1. Latest Scientific Advancements

Recent scientific research on industrial hemp has increasingly focused on the detailed characterization and optimization of its bioactive compounds, particularly cannabinoids, phenolic compounds, and terpenes. Leonard et al. [128] and Benkirane et al. [129] demonstrated that advances in analytical techniques, such as HPLC-DAD and mass spectrometry, enable precise identification and quantification of hemp-derived phytochemicals, especially in seeds and seed by-products. These studies highlighted the strong antioxidant and anti-inflammatory potential of hemp phenolics, reinforcing the plant’s growing relevance in functional food and nutraceutical development. At the molecular level, Shiels et al. [74] reported that genomic and biotechnological approaches have substantially advanced the understanding of cannabinoid biosynthesis pathways. In particular, genome-editing technologies, including CRISPR-based systems, are being explored to regulate cannabinoid composition, enhance stress tolerance, and stabilize agronomic traits in hemp varieties. This line of research reflects a clear shift from descriptive phytochemistry toward targeted metabolic and genetic optimization.

In contrast, current research on tobacco has expanded beyond nicotine to encompass a broader spectrum of phytochemicals, including polyphenols and minor alkaloids with potential pharmaceutical relevance [43,130]. Hong et al. [65] emphasized that tobacco remains an important model species in plant molecular biology, while biomedical research continues to focus on the toxicological and neurological effects of tobacco smoke constituents, underlining the persistent public health implications of tobacco-derived compounds.

### 12.2. Technological Innovations

Technological innovation has played a crucial role in shaping modern hemp and tobacco research. Leonard et al. [128] described significant progress in CBD extraction technologies, including supercritical CO_2_ extraction and optimized solvent-based methods, which improve extraction efficiency, product purity, and environmental sustainability. These advances are essential for standardizing hemp-derived products and ensuring reproducibility in pharmaceutical and food applications. Plant breeding and biotechnology represent another rapidly developing research area. According to Borin et al. [131], marker-assisted selection and modern breeding strategies are increasingly applied in hemp to control cannabinoid ratios, reduce THC content, and improve fiber or seed quality for industrial purposes. More recently, Shiels et al. [74] highlighted the growing role of genome-editing technologies in accelerating these breeding objectives. In tobacco, breeding strategies have similarly evolved to integrate agronomic and biochemical targets. Katatwile [132] reported that tobacco improvement programs increasingly focus on reduced nicotine content, modified alkaloid profiles, and improved curing efficiency, driven by regulatory constraints and environmental considerations. From an industrial perspective, Peev [133] noted that hemp has benefited from innovations in fiber processing and retting technologies, enabling its use in construction materials, insulation, and bio-composites, whereas technological developments in tobacco cultivation continue to be associated with considerable environmental impacts [132].

### 12.3. Potential Emerging Applications and Research Gaps

Emerging applications of hemp are increasingly interdisciplinary, spanning functional foods, pharmaceuticals, sustainable construction materials, and bio-based composites. Leonard et al. [128] demonstrated that hemp seeds and seed by-products represent valuable sources of high-quality protein, dietary fiber, and antioxidant compounds, while Peev [133] emphasized the growing importance of hemp fibers as sustainable alternatives to synthetic materials. Muhit et al. state that hemp-based materials have gained increasing attention in sustainable construction due to their favourable thermal properties, renewable origin, and potential to reduce the overall carbon footprint of buildings [134]. Industrial hemp represents a promising renewable feedstock for sustainable bioproducts and circular bioeconomy applications due to the wide range of valuable materials that can be obtained from its biomass [135,136]. Hemp-derived activated carbon has shown strong potential as a cost-effective and high-performance electrode material for supercapacitor applications [137]. Despite these advances, important research gaps remain regarding long-term safety, bioavailability of hemp-derived compounds, and the harmonization of regulatory frameworks across regions. In tobacco research, emerging applications are more controversial but scientifically relevant. Zhang et al. [130] described the increasing use of tobacco as a biotechnological platform for the production of vaccines and recombinant proteins, leveraging its well-characterized genetics and high biomass yield. However, ethical, environmental, and health-related concerns continue to limit the broader acceptance of such applications. Moreover, tobacco stems can be converted into bio-oil with significant termiticidal and antifeeding activity, indicating their potential as a source of natural insecticides [138].

Across both species, a major research gap lies in the integration of omics-based approaches with agronomy and environmental science. West and Cox [139] emphasized the need for a more holistic understanding of how cultivation practices, climate change, and post-harvest processing influence metabolite profiles and biological activity. Addressing these gaps will be essential for the sustainable exploitation of hemp’s beneficial properties while mitigating the well-documented risks associated with tobacco use. At the same time, emerging applications of tobacco-derived compounds in medicine and biotechnology illustrate a gradual shift from traditional consumption toward controlled and specialized uses [140].

## 13. Conclusions

Industrial hemp (*C. sativa* L.) and tobacco (*N. tabacum* L.) represent two plant species with exceptionally long histories of human use, whose roles have evolved from traditional, ritual, and medicinal practices to modern industrial, pharmaceutical, and economic applications. The key findings highlight that industrial hemp is a multifunctional crop rich in bioactive compounds, nutritionally valuable seeds, and sustainable fibers, while tobacco is characterized by a complex chemical composition dominated by nicotine and other alkaloids with well-documented physiological effects. Although both plants have been historically used as medicinal resources, contemporary research clearly distinguishes hemp as a crop with broad positive applications, whereas tobacco is primarily associated with health risks despite its continued relevance in pharmacological and biotechnological research. The practical and societal implications of these findings are significant. Industrial hemp has gained renewed importance due to its sustainability, low environmental impact, and versatility in food, construction, textile, and pharmaceutical industries, positioning it as a key crop within the bioeconomy and circular production systems. In contrast, tobacco remains economically important in many regions, providing livelihoods and contributing to national economies, yet its societal burden through tobacco-related diseases necessitates strict regulation and public health interventions.

Future research should focus on optimizing industrial hemp cultivation, improving the extraction and standardization of bioactive compounds, and further evaluating the long-term safety and efficacy of hemp-derived products. In the case of tobacco, research priorities should include harm-reduction strategies, alternative non-combustible uses, and the exploration of tobacco as a biofactory for pharmaceutical compounds rather than a consumer product. Generally, a balanced, science-based approach is essential to harness the benefits of these historically significant plants while minimizing their risks, ensuring that their transition from tradition to modern application aligns with sustainability, public health, and societal well-being.

## Figures and Tables

**Table 1:** Comparison of Tobacco Alkaloids and Cannabis Cannabinoids.

Feature	Tobacco (Nicotine/Alkaloids)	References	Cannabis (Cannabinoids)	References
Primary Target	Nicotinic acetylcholine receptors (nAChRs) in the brain.	[65]	CB1 and CB2 receptors of the endocannabinoid system.	[66,67]
Addictive Potential	High, primarily driven by nicotine and potentially enhanced by MAO inhibitors.	[68]	Varies; potential for cannabis use disorders, though often used for relief of withdrawal symptoms from other substances.	[66]
Common Smoke Components	Both contain carcinogens, ammonia, hydrogen cyanide, and particulate matter (PM).	[69]	Marijuana smoke often has higher levels of ammonia (up to 20-fold) and particulate matter than tobacco.	[70]
Antioxidant Activity	Less emphasized in primary alkaloids.	[71]	THC and CBD exhibit antioxidant activity comparable to vitamins E and C.	[72]

**Table 2:** Comparative overview of the botanical, agronomic, historical, and phytochemical characteristics of industrial hemp and tobacco.

Aspect	Industrial Hemp (*C. sativa* L.)	Tobacco (*N. tabacum* L.)	Main Comparative Feature
Plant family	Cannabaceae	Solanaceae	Taxonomically unrelated crops with markedly different morphology and chemical profiles
Growth habit	Annual, usually dioecious; rapid vegetative growth; strong fibrous stem	Annual or short-lived perennial; erect stem with large leaves	Hemp is mainly biomass-, fibre-, and seed-oriented; tobacco is primarily leaf-oriented
Typical plant height	Approximately 2–5 m, depending on genotype and conditions	Commonly around 1.5–2.0 m in commercial types	Hemp generally develops greater stem biomass
Root system	Deep and branched root system with numerous lateral roots	Well-developed root system, but crop value is concentrated in the leaves	Hemp root development contributes to soil exploration and structural effects
Optimal temperature	Approximately 16–27 °C	Approximately 20–30 °C	Hemp is generally adapted to more temperate conditions, while tobacco prefers warmer conditions
Soil requirements	Deep, well-drained soils; neutral to slightly alkaline pH	Well-drained, fertile soils with adequate N, P, and K	Tobacco generally requires more intensive nutrient management
Crop inputs	Relatively low pesticide requirements; dense stands can suppress weeds	Often requires intensive fertilization and pesticide protection	Hemp generally has lower external-input requirements
Main harvested organs	Stems, fibres, seeds, oil, flowers and other biomass fractions	Primarily leaves	Hemp permits broader whole-plant utilization
Traditional uses	Fibre, ropes, textiles, paper, food, oil, traditional medicine, and ritual uses	Ritual, ceremonial and medicinal use, followed by smoking, chewing and other tobacco uses	Both have long cultural histories, but their modern uses have diverged substantially
Dominant bioactive compounds	Cannabinoids, including CBD, THC/THCA, CBG and CBC; terpenes, phenolics and flavonoids	Nicotine and other alkaloids, including nornicotine, anatabine and anabasine; phenolics, flavonoids and terpenes	Cannabinoids characterize hemp, whereas alkaloids, particularly nicotine, dominate tobacco
Main biochemical relevance	Nutritional and functional compounds; antioxidant and potentially pharmacologically active constituents	Nicotine-related neuroactivity and dependence; additional phytochemicals with diverse biological activities	Chemical composition strongly determines their contrasting modern applications and safety profiles

**Table 3:** Neurobiological Mechanisms and Disease-Related Effects of Selected Tobacco Smoke Constituents.

Smoke Component	Mechanism	Disease State Affected	References
Nicotine	nAChR activation	Cognitive improvement	[82]
Nicotine	↓ α-synuclein fibril formation: (cognition ↑)	Parkinson’s disease	[83]
Nicotine	↓ Amyloid ß-peptide aggregation: (cognition ↑)	Alzheimer’s disease	[84]
Nicotine	Cognition ↑	Schizophrenia	[85]
Nicotine	Cognition ↑	Depression	[86]
Cotinine	↓ α-synuclein fibril formation, neuroprotection	Parkinson’s disease Alzheimer’s disease	[87]
Naphthoquinones	MAO inhibition, neuroprotection (nitric oxide control)	Parkinson’s disease	[88]
Harman/Norharman	MAO inhibition	Antidepressant	[89]
Harman/Norharman	MAO inhibition	Antianxiolytic	[89]
2,3,6-Trimethyl-1,4-naphthoquinone	MAO inhibition	Neuroprotection, Parkinson’s disease	[90]

Note: ↑ indicates an increase or improvement, whereas ↓ indicates a decrease, reduction, or inhibition.

**Table 4:** Health effects of tobacco use on different body systems.

Body System	Symptoms/Diseases	Reference
Cardiovascular	Poor blood circulation, numbness, high blood pressure, pressure in the chest, high cholesterol, myocardial infarction, stroke	[91,92]
Digestive	Gastritis, heartburn	[91,93]
Neurological	Depression, jittery, dizziness, giddy, “feel different,” automatic hand movement	[91,94]
Oral	Sore/dry throat, bad breath, loss of sense of taste, yellow teeth, gum disease	[91,95,96]
Reproductive	Erectile dysfunction, impact on pregnancy	[91,97,98]
Respiratory	Longer time to recover from a cold, quicker to get a cold, cough, pain in the lungs, asthma, chronic obstructive pulmonary disease (COPD), wheezing, trouble breathing/slower breathing, breathlessness or shortness of breath	[91,99]

**Table 5:** Comparative overview of health aspects, industrial applications, sustainability, and regulatory context of industrial hemp and tobacco.

Aspect	Industrial Hemp (*C. sativa* L.)	Tobacco (*N. tabacum* L.)	Comparative Interpretation
Nutritional use	Seeds and oil used as sources of protein, essential fatty acids, fibre and other nutrients	No established role as a conventional food crop	Food applications represent an important distinguishing feature of hemp
Pharmaceutical relevance	CBD and other constituents investigated or used for selected pharmaceutical applications	Controlled nicotine use mainly in smoking-cessation products; tobacco also serves as a research and biotechnology platform	Pharmaceutical use of hemp focuses mainly on selected bioactive compounds, while tobacco-derived nicotine is predominantly associated with dependence management
Major health considerations	Hemp seeds have nutritional value; CBD and other extracts require attention to dose, purity, labeling, drug interactions and unintended THC exposure	Tobacco consumption is strongly associated with addiction and major chronic health risks	Health implications differ substantially between whole hemp foods, hemp extracts, and tobacco consumption
Textile and fibre industry	Fibres used for textiles, ropes, paper and biodegradable materials	Limited conventional fibre application	Fibre production is one of hemp’s principal industrial advantages
Construction and biocomposites	Used for insulation, hemp-based composites, construction materials and automotive components	Limited application; some biomass residues are being investigated for alternative uses	Hemp has considerably broader established bio-based material applications
Food and cosmetics	Seeds, oil, flour, protein products and cosmetic formulations	No major conventional food use	Hemp has established food and cosmetic value chains
Alternative biomass uses	Biofuels, activated carbon, biocomposites and other circular-bioeconomy products	Stems, roots and processing residues may be explored for bioenergy, extraction of compounds and other specialized uses	Both have potential for biomass valorization, but hemp applications are more diversified
Agricultural sustainability	Rapid growth, relatively low pesticide demand, weed suppression, potential contribution to phytoremediation and bio-based production	Often associated with higher fertilizer and pesticide inputs and substantial crop and processing residues	Current literature generally positions hemp more strongly within sustainable and circular production systems
Main regulatory rationale	Primarily regulated through agricultural policy, drug-control legislation and THC limits	Primarily regulated from a public-health perspective	Regulatory objectives differ fundamentally between the crops
General cultivation status	Legal under defined THC limits in many jurisdictions; licensing and testing may be required	Cultivation generally legal but subject to national control and market regulation	Both are regulated crops, but for different reasons
Product regulation	Particularly complex for cannabinoids and CBD-containing foods, supplements and extracts	Extensive regulation of advertising, sale, taxation, age limits, packaging and public use	Hemp regulation focuses strongly on composition and product classification; tobacco regulation focuses strongly on reducing consumption and exposure

**Table 6:** Comparative regulatory framework for industrial hemp and tobacco in selected major markets.

Jurisdiction	Industrial Hemp	Tobacco	Key Regulatory Contrast	References
United States	Industrial hemp is currently defined as *Cannabis sativa* L. containing ≤0.3% Δ^9^-THC on a dry-weight basis. Production is regulated through federal, state, or Tribal plans and requires THC compliance testing. FDA restrictions remain important for CBD-containing foods and dietary supplements.	Tobacco products are regulated by the FDA with controls on manufacture, marketing, labelling, and retail sale; the federal minimum purchasing age is 21 years.	Hemp regulation combines agricultural, drug-control, and product-specific rules, whereas tobacco regulation is primarily focused on public health and consumption control.	U.S. Congress [110]; FDA [111]
European Union	Hemp varieties eligible under the CAP must contain ≤0.3% THC and certified seed of approved varieties must be used. Hemp-derived extracts and CBD-containing foods are additionally subject to EU food-safety and Novel Food requirements.	Tobacco manufacture, presentation, and sale are regulated under Directive 2014/40/EU; combined health warnings must cover 65% of the front and back of packages, together with other product and marketing restrictions.	Hemp is regulated mainly as an agricultural crop and according to cannabinoid/product status, whereas tobacco is regulated through harmonized public-health and market controls.	European Commission [112]; Directive 2014/40/EU [113]
China	Regulation is largely implemented at the provincial level. In Yunnan, industrial hemp is defined as cannabis containing <0.3% THC on a dry-weight basis, and licences are required for specified cultivation and processing activities.	Tobacco is subject to extensive state control, while China also implements tobacco-control measures under the WHO Framework Convention on Tobacco Control.	Hemp regulation is regionally differentiated and primarily linked to narcotics control and licensing, whereas tobacco operates under a highly centralized regulatory framework combined with public-health controls.	Yunnan Provincial Government [114,115]; WHO FCTC [112]
Canada	Industrial hemp is defined as cannabis containing ≤0.3% THC in the flowering heads and leaves. Most commercial cultivation and processing activities require a federal licence, and approved cultivars must be used. CBD extracted from flowering heads or leaves remains subject to the broader Cannabis Act framework.	Tobacco is regulated under the Tobacco and Vaping Products Act and related regulations, including standardized packaging and extensive health-warning requirements.	Both crops are federally regulated, but hemp is managed mainly through cannabis/hemp production controls, whereas tobacco regulation focuses on reducing health risks and product appeal.	Health Canada [116]

Note: Regulatory requirements may be supplemented by state, provincial, Member State, or local legislation and are subject to change. The information presented reflects the regulatory framework available in September 2026.

## Data Availability

The original contributions presented in this study are included in the article.

## Abbreviations

BC	Before Christ
CBC	Cannabichromene
CBD	Cannabidiol
CBG	Cannabigerol
CBN	Cannabinol
CB1	Cannabinoid receptor type 1
CB2	Cannabinoid receptor type 2
COPD	Chronic obstructive pulmonary disease
CRISPR	Clustered regularly interspaced short palindromic repeats
EU	European Union
FCTC	Framework Convention on Tobacco Control
HPLC-DAD	High-performance liquid chromatography with diode-array detection
MAO	Monoamine oxidase
nAChR	Nicotinic acetylcholine receptor
PM	Particulate matter
THC	Tetrahydrocannabinol
THCA	Tetrahydrocannabinolic acid
THCV	Tetrahydrocannabivarin
UN	United Nations
USA	United States of America
WHO	World Health Organization
Δ^9^-THC	Delta-9-tetrahydrocannabinol
Δ^9^-THCA	Delta-9-tetrahydrocannabinolic acid
